# Anodal cerebellar t-DCS impacts skill learning and transfer on a robotic surgery training task

**DOI:** 10.1038/s41598-023-47404-1

**Published:** 2023-12-20

**Authors:** Guido Caccianiga, Ronan A. Mooney, Pablo A. Celnik, Gabriela L. Cantarero, Jeremy D. Brown

**Affiliations:** 1 Laboratory for Computational Sensing and Robotics, Johns Hopkins University, Baltimore, 21218 USA; 2 Department of Physical Medicine and Rehabilitation, John Hopkins Medical Institute, Baltimore, 21218 USA; 3 Department of Mechanical Engineering, Johns Hopkins University, Baltimore, 21218 USA; 4 Present Address: Haptic Intelligence Department, Max Planck Institute for Intelligent Systems, Stuttgart, 70569 Germany; 5 Present Address: Shirley Ryan AbilityLab, Chicago, 60611 USA

**Keywords:** Biomedical engineering, Cerebellum, Neurophysiology, Surgery

## Abstract

The cerebellum has demonstrated a critical role during adaptation in motor learning. However, the extent to which it can contribute to the skill acquisition of complex real-world tasks remains unclear. One particularly challenging application in terms of motor activities is robotic surgery, which requires surgeons to complete complex multidimensional visuomotor tasks through a remotely operated robot. Given the need for high skill proficiency and the lack of haptic feedback, there is a pressing need for understanding and improving skill development. We investigated the effect of cerebellar transcranial direct current stimulation applied during the execution of a robotic surgery training task. Study participants received either real or sham stimulation while performing a needle driving task in a virtual (simulated) and a real-world (actual surgical robot) setting. We found that cerebellar stimulation significantly improved performance compared to sham stimulation at fast (more demanding) execution speeds in both virtual and real-world training settings. Furthermore, participants that received cerebellar stimulation more effectively transferred the skills they acquired during virtual training to the real world. Our findings underline the potential of non-invasive brain stimulation to enhance skill learning and transfer in real-world relevant tasks and, more broadly, its potential for improving complex motor learning.

## Introduction

Throughout the study of human and animal movement behavior, scientist have tried to classify and empirically delineate the different mechanisms of motor learning. The cerebellum has demonstrated a critical role in error-based learning through the development of forward internal models (sensory-motor maps) that are updated in accordance with sensory prediction errors. Such prediction errors provide vectorial information (e.g., magnitude and direction) on how to adjust the subsequent movement to achieve a successful motor action^1^. Therefore, error signals facilitate the update and refinement of the internal representations of the environment or body dynamics^2,3^.

Non-invasive brain stimulation (NIBS) is a tool that has been widely used in attempts to augment motor learning^4–6^. One form of NIBS called transcranial direct current stimulation (tDCS) consists of applying constant electric current into specific areas of the brain^7^ allowing for the investigation of physiological, functional, and behavioral reactions^8–11^. The cerebellum has been specifically targeted during several motor learning studies^12–15^. Through the application of anodal tDCS to the cerebellar cortex (CB-atDCS), Purkinje cells are thought to be activated, thereby inhibiting the excitatory connections to the primary motor cortex (M1). We think the effect of tDCS may be linked to Purkinje cells as they are the primary cells which have connections to cerebellar nuclei, which output to M1. However, we cannot exclude effects on other cells within the cerebellar cortex (e.g. inhibitory neurons, which also play an important role in motor control and learning). As a consequence, CB-atDCS has the potential to modulate the cerebellum-M1 interconnection and affect behavioral modifications during the execution of error-based motor learning tasks. Even though the specific neurophysiological mechanisms characterizing cerebellum and M1 are still only partially understood, the selective application of NIBS during tailored motor learning experiments is gradually leading to the disentanglement of their individual roles during the acquisition of real-world skills^1,16,17^.

Anodal tDCS stimulation over the ipsilateral cerebellum has been shown to augment online skill acquisition during a sequential visual isometric pinch force task^15^, and increase adaptation rates during a screen cursor rotation task^14^. Furthermore, CB-atDCS led to increased error-dependent learning and adaptation in a force-field reaching task^18^. Despite their promising results, these experiments utilized non-ecological, tightly controlled tasks with limited complexity. Therefore, the learned skills can be difficult to link to a meaningful real-world application. Likewise, to the best of our knowledge, no prior work has investigated the effects of CB-atDCS on skill generalization and context transfer.

In this work, we investigate the skill learning and skill transfer effects of CB-atDCS during complex visuomotor task execution in both real and virtual. The task, teleoperated needle insertion, requires participants to precisely guide a suture needle through an ideal planar trajectory. Using an open-source surgical robot, participants completed both a real-world and a virtual-reality version of the task in a crossover protocol. During the task, participants were shown visual feedback of task execution errors, which were measured as deviations from the ideal trajectory. Participants were also provided with auditory cues to enable compliance with prescribed task execution speeds. Utilizing a single within and between-participants study design, participants were randomized into four unique groups that varied in terms of stimulation protocol (CB-atDCS/Sham) and training environment presentation order (virtual-to-real/real-to-virtual).

We hypothesized that CB-atDCS applied during training would lead to measurable post-training behavioral changes with respect to sham stimulation (Sham). Furthermore, we hypothesized that virtual training would not be fully equivalent to real world training, in terms of both skill learning and transfer. After data collection, processing, and analysis, two unique findings emerged with respect to these two hypotheses. Our previous manuscript, Caccianiga et al.^19^, highlights our finding that real and virtual surgical training differ regarding skill learning and skill transfer. This result was based exclusively on findings from participants who received sham stimulation. Here, we highlight our other finding, pertaining to the overall impact of brain stimulation on complex visuomotor skill execution by considering the results of participants who received CB-atDCS stimulation and those who received sham stimulation in both the virtual-to-real and real-to-virtual training environments.

## Methods

### Participants

This study was approved by the Johns Hopkins School of Medicine Institutional Review Board (IRB: study #00077792). All reported methods were carried out following the IRB guidelines and regulations. 36 able-bodied participants were recruited for the study (17 females and 19 males; mean age 27 ± 4.1 years). Informed consent was obtained from all subjects prior to the experiment. 33 participants reported being right-hand dominant, as assessed using the Edinborough Handedness Survey. Three participants reported being left-hand dominant and performed the experiment on a mirrored setup. Among the participants, 12 had medical backgrounds, however, no participants had prior experience with laparoscopy, robotic surgery, or any other teleoperation device. Given the nature of the experimental task, we assume educational background, gender, and handedness have no relevant impact on the study results. All participants came in for a single session (approximately 120 min) during which they were asked to perform a surgical training task in either a real or virtual training environment and then switch to the opposite training environment. In a double-blind fashion, participants received either real or sham cerebellar stimulation during training.

**Figure 1:**
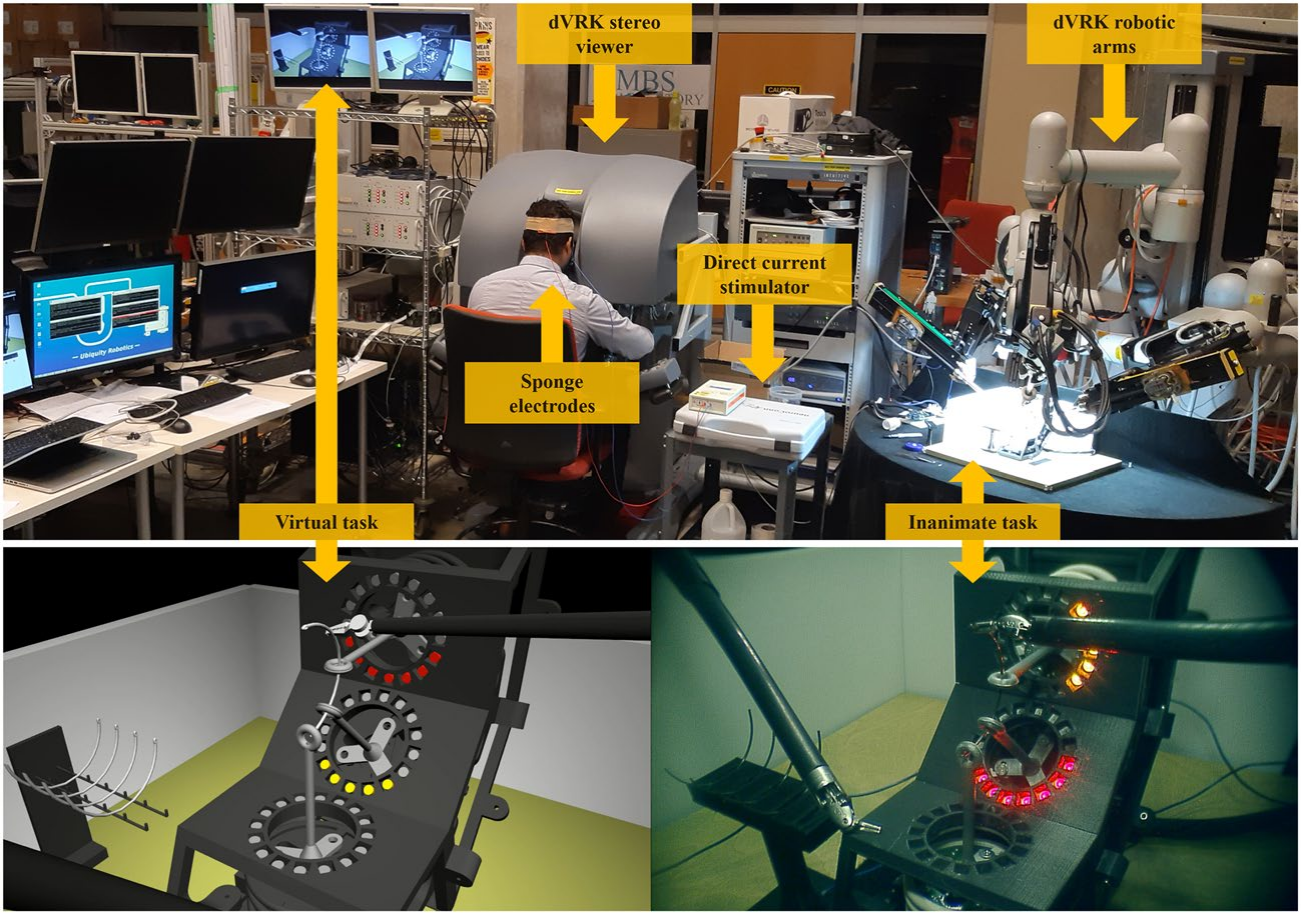
Top—the experimental setup. The participant sits at the surgical robot console while receiving NIBS. The robot is composed of two main components. First, the surgeon’s console (dVRK stereo viewer), where the participant remotely controls the surgical instruments with two hand manipulators and an immersive stereoscopic view of the operation site. Second, the patient side console (dVRK robotic arms) where the surgical instruments are deployed and the stereo image of the scene is captured. Bottom—the Virtual (left) and Inanimate (right) Enhanced Needle Driving (END) platforms as seen from the dVRK stereo viewer. Proportions, perspective, and background are accurately reproduced between the two training platforms. Two surgical instruments are teleoperated to drive the curved needle through three rings. Visual feedback of each ring displacement is provided through a ring of RGB LEDs. Feedback changed in terms of position of the activated LED on the LEDs ring (displacement direction), color of the activated LED (displacement intensity), and the number of LEDs activated (push/pull displacement).

### Experimental task

For the complex motor learning task, we utilized the Enhanced Needle Driving (END) platform, an experimental setup developed to allow direct comparisons between virtual reality and real-world inanimate surgical training^19^. Training experiments were performed using the da Vinci Research Kit (dVRK), an open-source telerobotic system derived from the first generation da Vinci Surgical System^20^. The END training task involved driving a curved surgical needle (1/2 round, 20 mm radius) through three rings (2 mm radius) distributed at 45 degree increments inside the vertical plane. The END platform showed multidimensional visual feedback of the needle trajectory error through a ring of LED lights. The visual feedback displayed the lateral displacement direction by turning on one of the 24 LEDs of the LED ring (like the hand of a clock). Additionally, the lateral displacement intensity was mapped to the color of the selected LED (e.g. red or yellow). Furthermore, in case of both lateral and axial displacement (push/pull), the number of activated LEDs would increase according to the axial displacement intensity. A real sensorized END platform (Inanimate) and identical simulated END platform (Virtual) were developed to support investigations into context-specific skill acquisition (Fig. 1). Complete details of the experimental task and telerobotic platform can be found in^19^.

### Cerebellar stimulation

Cerebellar stimulation was delivered using a neuroConn DC-Stimulator (Neurocare group AG, 2021) using two 25 cm$^2$ sponge electrodes soaked in saline solution. A cerebellar montage was used with the anode centered over the cerebellum (3 cm lateral to the inion, ipsilateral to the user’s dominant hand) and the cathode electrode positioned in the central region of the ipsilateral cheek. This is the standard montage used for cerebellar tDCS^15,21^. The intensity of stimulation was ramped up to 2 mA at the beginning of the training phase. The stimulation intensity was set based on previous investigations reporting the utility and robustness of a 2 mA current flow across the cerebellum^15,22^ as well as other brain regions^23^. The stimulation protocol delivered 30 min of CB-atDCS while the Sham protocol delivered stimulation only for the first 30 s. The control unit was set in double-blind mode so that neither the trainee nor the investigator was aware of the actual level of current output. Prior to stimulation, participants were checked for any discomfort related to the electrodes’ setup.

**Figure 2:**
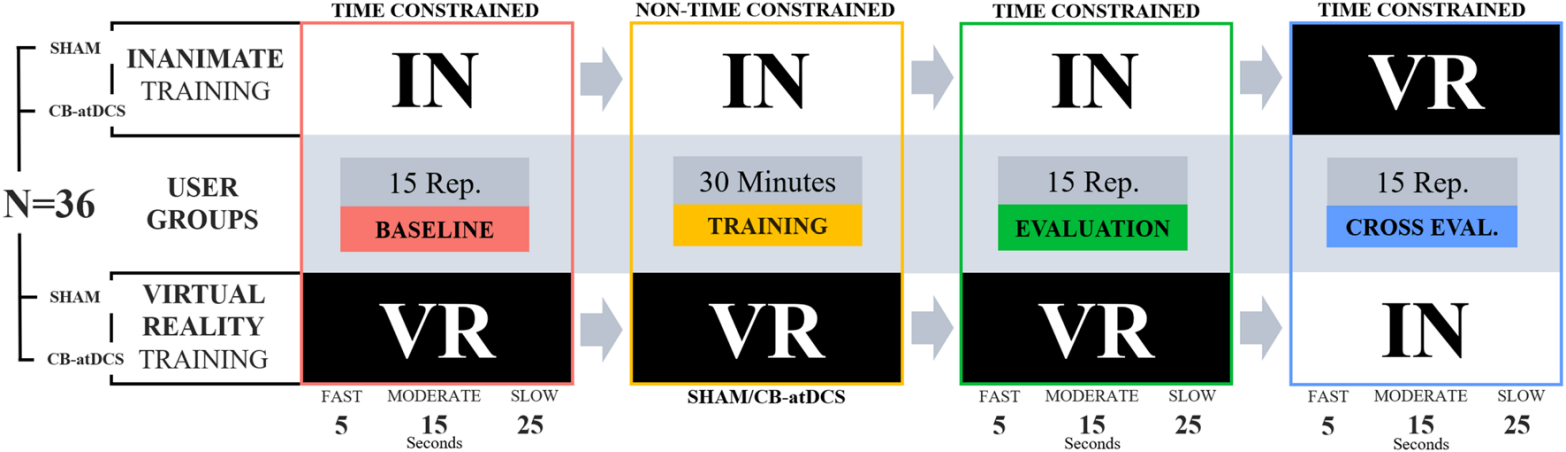
Overview of the study design. Participants were divided into four groups (N = 9 for each group) based on the training platform (Inanimate or Virtual) and stimulation (CB-atDCS or Sham). During the *Training* phase (yellow box), half of the participants received sham stimulation while the other received real CB-atDCS. Participants underwent three testing phases: *Baseline* (pre-training, shown in red), *Evaluation* (post-training, shown in green), and *Cross-evaluation* (opposite platform, shown in blue). For each of the testing phases, time constraints were introduced guiding the user towards a *Fast* (5 s), *Moderate* (15 s), or *Slow* (25 s) execution speed. Task executions during *Training* were not time constrained.

### Experimental design

Participants completed a tDCS eligibility survey regarding medical background, demographics, and handedness. They were then familiarized with the robotic platform (dVRK) and received an overview of the experimental needle driving task. Participants were instructed on the functioning principle of the visual feedback provided through the RGB LED lights around each ring. Users were then randomly assigned to the following four groups: 1) sham stimulation on the Virtual END platform (*Virtual-Sham*, 4 females and 5 males, mean age 27 ± 3.7 years, 1 left-handed, 2 with medical background); 2) real stimulation on the virtual END platform (*Virtual-Stim*, 5 females and 4 males, mean age 29 ± 5.2 years, 1 left-handed, 4 with medical background); 3) sham stimulation on the Inanimate END platform (*Inanimate-Sham*, 4 females and 5 males, mean age 27 ± 3.4 years, 0 left-handed, 4 with medical background); 4) real stimulation on the Inanimate END platform (*Inanimate-Stim*, 3 females and 6 males, mean age 27 ± 4.7 years, 1 left-handed, 2 with medical background). During the training phase, participants in the Sham groups received 30 s of CB-atDCS stimulation, whereas participants in the Stim groups received 30 min of CB-atDCS stimulation (described below).

The experiment consisted of four distinct phases: *Baseline*, *Training*, *Evaluation*, and *Cross-evaluation*. During the *Baseline* phase, participants’ initial skill level was evaluated with 15 task trials (a trial is defined as a completed single needle insertion). 15 trials was chosen here based on prior tDCS studies^15,24^ and a desire to minimize significant skill development in the task. During the *Training* phase, participants performed trials over the course of 30 min, receiving either CB-atDCS or sham stimulation. During the *Evaluation* phase, participants performed 15 trials of the task on the same platform they trained on. During the *Cross-evaluation* phase, participants repeated their post-training evaluation on the opposite platform with respect to the one used during the *Baseline*, *Training*, and *Evaluation* phases. Overall, each group trained exclusively on one type of platform (Virtual or Inanimate) and was made aware of the other type of platform only at the time of the second post-training evaluation (*Cross-evaluation*).

During the *Training* phase, users were not time-constrained and therefore free to decide their own trade-off between speed and accuracy. During the testing phases (*Baseline*, *Evaluation*, and *Cross-evaluation*), users were instructed to follow three different prescribed task execution speeds: *Fast* speed—5 s, *Moderate* speed—15 s, and *Slow* speed—25 s. These execution times were designed to sample participants’ performance at three distinct points on the speed-accuracy tradeoff function^25^ and were based on empirical task execution times of an experienced user. Auditory and verbal cues were provided for time keeping. They consisted of auditory beeps in one second increments and a verbal countdown of the time remaining in five second increments (e.g., “Fifteen” beep beep beep beep “Ten”). A graphical representation of the whole experimental protocol can be seen in Fig. 2.

Five task repetitions for each of the three task execution speeds were presented in a randomized order. An experimenter monitored the process, and whenever the participant exceeded a ±5 s interval from the prescribed time, the prescribed time was presented again on the following repetition. Given the propensity for participants to perform the task at the *Moderate* speed (15 s), the experimenter often asked participants to repeat the extreme speeds (5 and 25 s). This close monitoring of the execution time allowed the experimenter to guide the participant through an evenly distributed sampling across the speed-accuracy space.

### Performance metrics and statistical analysis

For each needle insertion, we measured the Euclidean distance $D_{i}(n)$ of displacement for each ring with respect to its resting position. This displacement measure was averaged for each trial and summed across the three rings as follows:

1 $$\begin{aligned} \text {Mean Ring Displacement}=\sum _{i=1}^{3}\left( \frac{\sum _n{D_i(n)}}{max(n)}\right) \end{aligned}$$

Where *i* represents the ring number, and *n* the specific data point while sampling at 60Hz. The *Mean Ring Displacement* metric is therefore a single number describing the average displacement error (mm) for each trial (see^26^ for complete details). This performance metric was chosen as it accounts for both needle trajectory error and execution speed.

The three speed classes (*Fast, Moderate, Slow*), originally defined by the requested time, were redefined through clustering analysis based on participants’ actual completion time. The resulting three effective task execution times are created by splitting the actual completion time distributions at the 33rd (11.65 s) and 66th (18.13 s) percentiles. This newly defined effective task execution time allowed for statistical comparisons over almost even samples distributions (*Fast*: less than 11.65 s, # samples = 550; *Moderate*: between 11.65 and 18.13, # samples = 549; *Slow*: more than 18.13 s, # samples = 549).

Considering the large behavioral variability that was allowed (and observed) between participants during the *Training* phase, we will not perform any statistical analysis on the *Training* phase data. We are therefore not able to analyze the effect of CB-atDCS during the *Training* phase.

Using a Linear Mixed Model (LMM, Gaussian distribution) we defined *Mean Ring Displacement* as dependent variable, and Speed (*Fast, Medium, Slow*), Phase (*Baseline, Evaluation, Cross-evaluation*), Platform (Virtual, Inanimate), and Protocol (Sham, CB-atDCS) as independent variables. The LMM accounted for the repeated measures design of the experiment and supported modeling of all the possible interactions between the independent variables. The model constructed on the 4-way interaction of all the independent variables (Protocol:Platform:Phase:Speed) resulted in the best performing model (see Table 1). In analyzing the data, we noticed the distribution of the *Mean Ring Displacement* followed a log-based skew. This characteristic often occurs in unsigned error-based metrics, which show a high density left-skew in the proximity of zero. We therefore applied a Log10 transformation to our dataset. After such transformation, the residuals of the model pass the Shapiro-Wilk test of normality (*p* = 0.294). Post-hoc tests were then directly performed on the LMM estimates using simultaneous t-tests with Satterthwaite’s method. A Bonferroni correction was applied to different groups of simultaneous linear hypotheses, as distributed in Tables 2 and 3. All statistical analyses were performed using the lme4 package in R version 3.5.3 (The R Foundation for Statistical Computing, Vienna).

**Table 1:** Linear Mixed Models performance comparison investigating the effects of Intercept (Z), Stimulation (S), Platform (P), Phase (F), Speed (T), and their two-, three-, and four-way interactions.

Model	Df	AIC	logLik	*p*-value
Z		912.79	− 453.39	
S	1	912.41	− 452.21	0.123
P	0	913.42	− 452.71	
F	1	712.27	− 351.13	< **0.001**
T	0	552.35	− 271.18	
S:P	1	913.95	− 450.98	1.000
S:F	2	707.24	− 345.62	< **0.001**
S:T	0	554.96	− 269.48	
P:F	0	706.80	− 345.40	
P:T	0	553.63	− 268.82	
F:T	3	254.61	− 116.30	<**0.001**
S:P:F	3	702.60	− 337.30	1.000
S:P:T	0	560.78	− 266.39	
S:F:T	6	253.74	− 106.87	<**0.001**
P:F:T	0	232.04	− 96.02	
S:P:F:T	18	240.45	− 82.22	0.068

*p*-values are referring to comparison with the respective previous row. Where the number of parameters is equal between two rows (Df  =  0), no p-value is computed.

## Results

### Model construction

Table 1 shows a performance comparison of the intermediate interaction models starting from the intercept only (Z) up to the final 4-way interaction model we used to produce the post-hoc results (S:P:F:T). Both Phase (F) and Speed (T) had a significant fixed effect on the dependent variable *Mean Ring Displacement* with respect to the intercept-only model (Z). All the 2-way interactions are statistically significant with respect to the single fixed effect model, apart from the one between Stimulation (S) and Platform (P). The two-way interaction between Phase (F) and Speed (T) is significantly stronger than the other 2-way interactions. Adding Stimulation (S) or Platform (P) to form a three-way interaction with Phase (F) and Speed (T) both leads to significantly better fitting models. The 4-way interaction (S:P:F:T) produces a slight performance increase with respect to the 3-way models, yet not a statistically significant increase. We therefore chose the 4-way interaction model since it produced the second-lowest Akaike Information Criterion (AIC) and the lowest log-likelihood (logLik) values, plus, it allowed for consideration of all four fixed effects simultaneously. This model was chosen for its ability to allow post-hoc hypothesis testing at the lower clustering level (Stimulation:Platform:Phase:Speed).

### Performance analysis

Overall, participants in all four groups were able to successfully complete the task. Despite not being constrained to a certain number of trials repetitions during the *Training* phase, we observed that participants completed a similar number of trials in each of the four groups (Virtual-Sham: tot = 299, avg = 33.22, std = 8.71; Virtual-Stim: tot = 336, avg = 37.33, std = 5.45; Inanimate-Sham: tot = 332, avg = 36.88, std = 2.75; Inanimate-Stim: tot = 352, avg = 39.11, std = 1.26, where tot is the total number of trials cumulatively—sum across number of trials of each participant of a group—performed by each participant group during *Training*).

To fully characterize the potential effect of CB-atDCS, we report the results while tracking the performance of each participant along the three testing phases of the trial (Baseline, Evaluation, Cross-evaluation). These within-group analyses (Tables 2, 3) are sensitive to individual skill development, as they compute deltas (relative) performance changes within each participant group. For completeness, we also report the between-group comparisons (Table 4) for the Sham and CB-atDCS groups.

#### Skill learning

**Figure 3:**
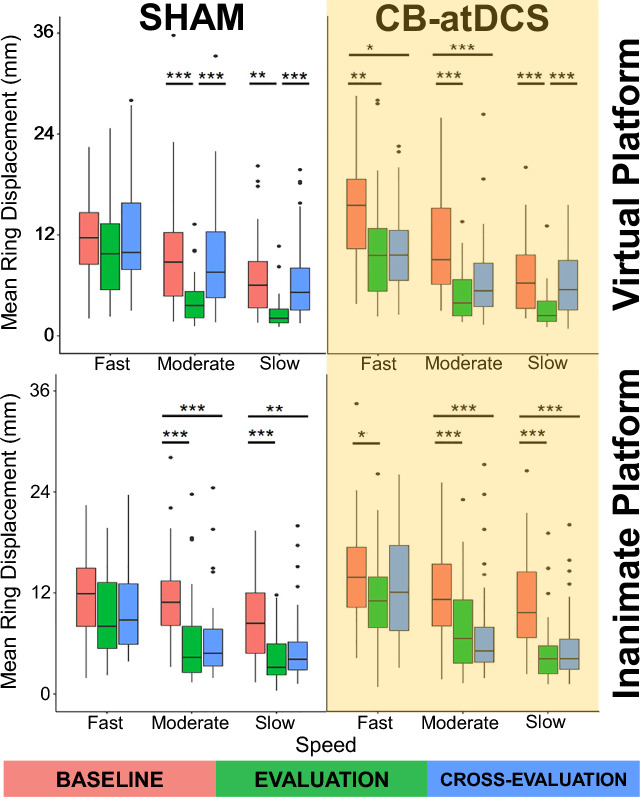
Overview of the four groups performances. The *Mean Ring Displacement* metric (needle trajectory error) is shown for the three evaluation phases (*Baseline*, *Evaluation*, and *Cross-evaluation*) at a specific task execution speed (*Fast, Moderate,*and *Slow*). *Skill learning:* participants in all four groups significantly improved their performance (lower error) from *Baseline* (pre-training) to *Evaluation* (post-training) at both the *Moderate* and *Slow* speeds. Furthermore, groups receiving CB-atDCS significantly improved post-training performance (*Evaluation*) also at the *Fast* speed, whereas groups receiving Sham did not. *Skill transfer:* both groups trained on the Inanimate platform kept the performance when transferring to the Virtual platform (*Cross-evaluation*) at *Moderate* and *Slow* speed (comparable error). The group trained on the Virtual platform receiving Sham did not transfer the performance when moving to the Inanimate platform (*Cross-evaluation*) at any speed (increased error), while the group receiving CB-atDCS did transfer performance at *Fast* and *Moderate* speeds. Statistical significance convention: $\star$ *p* < 0.05; $~\star \star$ *p* < 0.01; $~\star \star \star$ *p* < 0.001.

Participants in all four groups significantly improved their performance from *Baseline* (pre-training) to *Evaluation* (post-training) at both the *Moderate* and *Slow* speeds (*p*<0.05). For the *Fast* speed, groups receiving sham stimulation (Virtual-Sham, Inanimate-Sham) demonstrated no significant improvements (*p* > 0.05) from *Baseline* to *Evaluation*. In contrast, groups receiving CB-atDCS (Virtual-Stim, Inanimate-Stim) did have a statistically significant improvement in error between *Baseline* and *Evaluation* at the *Fast* speed (*p*<0.05). See Fig. 3, Table 2, and Table 3 for details.

Importantly, participants in all the four groups demonstrated comparable performance at *Baseline* with no statistically significant difference at *Baseline* between groups on the same platform or on different platforms (*p* > 0.05). Table 4 reports the between-group comparisons for Sham and CB-atDCS for *Baseline* and *Evaluation*. Even though no statistically significant difference was found between Sham and CB-atDCS at *Evaluation*, overall, our findings suggest that, with comparable initial skill, groups receiving CB-atDCS significantly improved Skill Learning at the *Fast* speed whereas groups receiving Sham did not.

#### Skill transfer from real to virtual environment

Participants in the Inanimate-Sham and Inanimate-Stim groups demonstrated no significant difference in performance between the *Evaluation* phase (post-training) on the Inanimate END platform and the *Cross-evaluation* phase on the Virtual END platform at *Moderate* and *Slow* speeds (*p* > 0.05). Likewise, performance in the *Cross-evaluation* phase was significantly higher (lower error) than *Baseline* (*p*<0.05) for the *Moderate* and *Slow* speeds. At the *Fast* speed, however, there was no significant difference between performance in the *Cross-evaluation* phase and performance in *Baseline* (*p* > 0.05).

#### Skill transfer from virtual to real environment

Participants in the Virtual-Sham group, significantly decreased their performance (higher errors) (*p*<0.05) between the *Evaluation* phase on the Virtual END platform and the *Cross-evaluation* on the Inanimate END platform at *Moderate* and *Slow* speeds. Likewise, performance in the *Cross-evaluation* phase was not significantly different from *Baseline* (*p* > 0.05) at the *Fast*, *Moderate*, and *Slow* speeds. Conversely, participants in the Virtual-Stim group, demonstrated no significant difference in performance between the *Evaluation* (virtual) phase and the *Cross-evaluation* (inanimate) phase at *Fast* and *Moderate* speeds (*p* > 0.05). Likewise, performance in the *Cross-evaluation* phase was significantly higher (lower errors) than *Baseline* (*p*<0.05) at the *Fast* and *Moderate* speeds. At the *Slow* speed, there was no significant difference in *Cross-evaluation* and *Baseline* performance for the Virtual-Stim group (*p* > 0.05). See Fig. 3, Table 2, and Table 3 for detailed results.

Additionally, Table 4 reports the between-group comparisons for Sham and CB-atDCS for *Cross-evaluation*. Even though no statistically significant difference was found between Sham and CB-atDCS at *Cross-evaluation*, overall, our findings suggest that, with comparable post-training performance, groups receiving CB-atDCS achieved significant skill transfer at the *Fast* and *Moderate* speeds while groups receiving sham stimulation did not.

**Table 2:** The *MeanRingDisplacement* metric is compared within the two participant groups trained in the Virtual platform (Virtual-Sham, Virtual-Stim). Skill learning—*Baseline* to *Evaluation* (EV-BL) and skill transfer—*Baseline* to *Cross-evaluation* (CR-BL), are shown for the *Slow, Moderate*, and *Fast* speeds.

Group	Virtual-Sham		Virtual-Stim	
Skill learning	*p-value*	Effect size	*p-value*	Effect size
(EV-BL)Slow	<**0.001**	1.42	<**0.001**	1.31
(EV-BL)Moderate	<**0.001**	1.23	<**0.001**	1.49
(EV-BL)Fast	0.629	0.45	**0**.**008**	0.70

Group	Virtual-Sham		Virtual-Stim	
Skill transfer	*p-value*	Effect size	*p-value*	Effect size
(CR-BL)Slow	1.000	0.07	1.000	0.25
(CR-BL)Moderate	1.000	0.13	<**0.001**	0.99
(CR-BL)Fast	1.000	− 0.01	**0**.**018**	0.72
(CR-EV)Slow	<**0.001**	− 1.35	<**0.001**	− 1.01
(CR-EV)Moderate	<**0.001**	− 1.07	0.556	− 0.36
(CR-EV)Fast	0.365	− 0.48	1.000	− 0.08

Estimates are based on the $Log_{10}$ data. Effect size is reported as *Cohen’s d* ($|d|<0.2$ “negligible”, $|d|<0.5$ “small”, $|d|<0.8$ “medium”, otherwise “large”).

**Table 3:** The *MeanRingDisplacement* metric is compared within the two participant groups trained in the Inanimate platform (Inanimate-Sham, Inanimate-Stim). Skill learning—*Baseline* to *Evaluation* (EV-BL) and skill transfer—*Baseline* to *Cross-evaluation* (CR-BL), are shown for the *Slow, Moderate*, and *Fast* speeds.

Group	Inanimate-Sham		Inanimate-Stim	
Skill Learning	*p-value*	Effect size	*p-value*	Effect size
(EV-BL)Slow	<**0.001**	1.097	<**0.001**	1.453
(EV-BL)Moderate	<**0.001**	1.350	<**0.001**	0.804
(EV-BL)Fast	1.000	0.400	**0**.**01**	0.621

Group	Inanimate-Sham		Inanimate-Stim	
Skill Transfer	*p-value*	Effect size	*p-value*	Effect size
(CR-BL)Slow	**0**.**003**	0.724	<**0.001**	1.225
(CR-BL)Moderate	<**0.001**	1.268	<**0.001**	0.932
(CR-BL)Fast	1.000	0.247	1.000	0.252
(CR-EV)Slow	0.074	− 0.413	0.299	− 0.229
(CR-EV)Moderate	1.000	− 0.247	1.000	0.054
(CR-EV)Fast	1.000	− 0.173	1.000	− 0.376

Estimates are based on the $Log_{10}$ data. Effect size is reported as *Cohen’s d* ($|d|<0.2$ “negligible”, $|d|<0.5$ “small”, $|d|<0.8$ “medium”, otherwise “large”).

**Table 4:** The *MeanRingDisplacement* metric is compared between the groups that received Sham and CB-atDCS stimulation. Comparisons are presented for *Baseline*, *Evaluation*, and *Cross-evaluation* and shown for the *Slow, Moderate*, and *Fast* speeds.

Group	Virtual		Inanimate	
Baseline	*p-value*	Effect size	*p-value*	Effect size
(Sham-Stim)Slow	1.000	− 0.139	0.889	− 0.441
(Sham-Stim)Moderate	1.000	− 0.306	1.000	− 0.029
(Sham-Stim)Fast	1.000	− 0.404	0.386	− 0.534

Group	Virtual		Inanimate	
Evaluation	*p-value*	Effect size	*p-value*	Effect size
(Sham-Stim)Slow	1.000	− 0.247	1.000	− 0.197
(Sham-Stim)Moderate	1.000	− 0.181	0.414	− 0.380
(Sham-Stim)Fast	1.000	− 0.105	1.000	− 0.215

Group	Virtual		Inanimate	
Cross-evaluation	*p-value*	Effect size	*p-value*	Effect size
(Sham-Stim)Slow	1.000	0.037	0.682	− 0.006
(Sham-Stim)Moderate	0.216	0.511	1.000	− 0.132
(Sham-Stim)Fast	1.000	0.313	0.618	− 0.522

Estimates are based on the $Log_{10}$ data. Effect size is reported as *Cohen’s d* ($|d|<0.2$ “negligible”, $|d|<0.5$ “small”, $|d|<0.8$ “medium”, otherwise “large”).

## Discussion

In this study, we investigated the effects of cerebellar stimulation delivered during training in a complex surgical visuo-motor task. We previously demonstrated that our feedback-augmented needle driving task engages error-driven learning and is capable of measuring significant performance changes in a single training session^19^. Therefore, we hypothesized that cerebellar anodal transcranial direct current stimulation (CB-atDCS) applied during training of our feedback-augmented needle driving task would lead to measurable post-training behavioral changes. Given the task completion time constraints introduced in each of the testing phases (*Baseline, Evaluation*, and *Cross-evaluation*), we systematically sampled performance across the speed-accuracy trade-off, reducing the motor learning process to a single dimensional feature^27^. As a result, direct quantitative comparisons on accuracy (at equivalent speeds) were possible across the dataset. Specifically, motor learning was evaluated in terms of *Skill Learning* (pre- to post-training) and *Skill Transfer* (post-training transfer from virtual to real task and vice-versa) at three different execution speeds (*Fast, Moderate*, and *Slow*). Our findings suggest that groups receiving CB-atDCS improved *Skill Learning* at the *Fast* speed, while groups receiving Sham did not. Additionally, with comparable post-training performance, groups receiving CB-atDCS achieved *Skill Transfer* at the *Fast* and *Moderate* speeds while groups receiving sham stimulation did not.

Our finding of improved skill learning at only the *Fast* speed for participants receiving CB-atDCS could be explained in the context of the cerebellum’s role in motor prediction and update^1,16^. Producing an accurate movement at *Fast* speed represented the most challenging and demanding aspect of the proposed task (following Fitt’s law) and, therefore, the most likely to benefit from NIBS stimulation. More specifically, constraining participants to execute the task at the *Fast* speed (5 s) forced them to perform the task execution in a more ballistic manner. In this context, participants relied less on concurrent visual feedback to minimize the error of the needle trajectory. Instead, participants had to rely on the accuracy of the internal representation of the environment and the task, and, therefore, feedforward mechanisms known to rely on cerebellar neural substrates^28^. Thus, in this specific context CB-atDCS shows a more prominent role, facilitating the learning and execution of fast yet accurate complex movements. This interpretation aligns with previous studies showing that tDCS improved shooting precision in ballistic sports like tennis or basketball^29–31^. Overall, our findings on skill learning also fit with recent research showing promising effects of tDCS compared to sham stimulation (mostly applied to M1 or the pre-frontal cortex) in the context of open^32^, laparoscopic^33–37^, robot-assisted^38^, and virtual reality^39^ surgical training.

In our previous analysis of the Sham dataset by itself^19^ we found that the skill transfer process was not bidirectional. While skills learned on the inanimate needle-driving task were successfully transferred to the Virtual End task, the converse was not true, skill learned on the virtual needle-driving task did not transfer to the Inanimate END task. The present analysis extends these findings by uncovering a potential role of CB-atDCS in improving skill transfer. Here we found that CB-atDCS during the inanimate needle-driving task in *Cross-evaluation* was significantly better than baseline performance on the Virtual END task, but not significantly different than *Evaluation* performance on the Virtual END task for the *Fast* and *Moderate* speeds. Stimulation of the cerebellum with tDCS may have created a more generalizable internal representation of the task and environment dynamics obtained during learning. This might have made it easier for participants to transfer their skill from a less realistic context (Virtual task) to the real world (Inanimate task). Several studies have been carried out, separately, on the effects of NIBS applied during virtual reality training^40–42^; and separately on the transfer of skills from the virtual to the real world context^43–48^. To the best of our knowledge, our study is the first to investigate the effect of NIBS on the bidirectional skill transfer between virtual and real-world training.

It is worth considering here that the observations made in this study are heavily influenced by the nature of the motor learning task. Due in part to the absence of haptic feedback, telerobotic surgery requires users to learn a control strategy that cannot rely innately on the availability of cutaneous and kinesthetic cues to close a sensorimotor loop. In addition, the particular needle driving task used in this study requires solving an inverse dynamics problem to restrict the six degrees of freedom of the needle to planar three degree of freedom movement. Thus, the observed findings regarding CB-atDCS indicate the utility of non-invasive brain stimulation on improving complex ecological motor learning tasks. Overall, we envision additional studies investigating various forms of real-world tasks augmentation. We hope our present work can serve as a starting point for future researchers hoping to push the forefront of brain stimulation utility across task complexity. To infer predictive validity of the enhanced training methodology reported here, future studies should include one or more subsequent skill transfer sessions where participants are asked to perform an actual surgical suturing task on soft tissue. Ad-hoc study protocols would have to be designed to extend our findings through time and surgical sub-procedures. Of key importance would be a systematic assessment of how task complexity impacts skill learning and transfer to better understand how different layers of skill complexity combine and how NIBS could affect such processes.

When validated in a larger sample, the results of these studies could have a significant impact on robotic surgery training programs. Enhancing skill transfer through non-invasive brain stimulation could speed up the training time and help shorten the overall robotic surgery learning curve. To bridge our experimental setting to a real world application, some practical challenges would have to be overcome. For example, the use of tDCS requires skilled personnel to monitor and maintain the right amount of moisture at the electrode-skin interface. In addition, the use of tDCS needs to be thoroughly weighed against other physiological factors that are common in medical training routines (e.g., discontinuous sleep cycles, medications, stimulants, cognitive load).

While the results of our study are very promising, there are a few limitations that merit highlighting for future research. First, despite our positive results, our sample size was relatively small. First, despite our positive results, our relatively small sample size and lack of an a priori power analysis limits the broad implications of our findings. Thus, we acknowledge that these results are more exploratory than definitive, and need to be robustly confirmed in follow-on studies that are prospectively designed according to standardized guidelines such as a randomized controlled trial. This limitation is especially relevant when considering the absence of statistically significant differences in our between-group comparison for the evaluation phases. Second, time constraints during the testing phases, while effective, were only able to guide the participant towards a generalized and not exact 25, 15, or 5 s execution speed. Furthermore, auditory and verbal timekeeping could have created time pressure on participants that may not have been experienced uniformly. In future studies, it may be worth investigating to what extent, if any, participants’ perception of the timing cues affected their ability to perform the task at that specified time. Likewise, since we limited the *Training* phase to 30 min (for stimulation consistency) and participants were free to move at their own selected speed during training, we were not able to control the number of task repetitions during the *Training* phase for each participant. This limited our ability to perform direct statistical comparisons across subject groups during the *Training* phase. To further optimize our protocol, we envision a more structured data acquisition during the *Training* phase, and the introduction of longitudinally delayed post-training tests to evaluate the effects of CB-atDCS over long-term skill retention. It would also be interesting to test different stimulation current intensities on separate control groups to investigate the amount of stimulation required to generate a behavioral change. Likewise, while we do have a sham condition in this study, we do not have control stimulation sites. Therefore, additional investigations could be conducted to investigate regional specificity.

## Conclusion

We found that cerebellar anodal transcranial direct current stimulation (CB-atDCS) applied during the training of a feedback-augmented needle driving task leads to measurable post-training behavioral changes both in terms of *Skill Learning* and *Skill Transfer*. The ability to boost real-world skill acquisition through non-invasive brain stimulation has implications to wide swath of visuo-motor learning tasks. In particular, when considering the portability of the CB-atDCS approach utilized here. Additionally, the present findings regarding skill transfer from the virtual to the physical domain has the potential to impact the field of robotic surgery training, as well as healthcare or other industrial applications that involve extensive training in simulated environments.

## Data Availability

The datasets generated during and/or analyzed during the current study are available from the corresponding author on reasonable request.
